# Editorial: Community series in unraveling the molecular mechanisms of cytokine signaling in regulating inflammatory diseases, volume II

**DOI:** 10.3389/fimmu.2026.1828794

**Published:** 2026-03-26

**Authors:** Jian Zheng, Huawei Mao, Wai Po Chong

**Affiliations:** 1 Department of Microbiology and Immunology, University of Louisville, Louisville, KY, United States; 2 Center for Predictive Medicine, University of Louisville, Louisville, KY, United States; 3 Department of Rheumatology and Immunology, Ministry of Education Key Laboratory of Major Diseases in Children, Beijing Children’s Hospital, Capital Medical University, National Center for Children’s Health, Beijing Key Laboratory of Precision Diagnosis and Treatment of Pediatric Immune Diseases, Beijing, China; 4 Department of Nephrology and Rheumatology, Children’s Hospital of Xinjiang Uygur Autonomous Region, Xinjiang Hospital of Beijing Children’s Hospital, Xinjiang, China; 5 School of Chinese Medicine, Hong Kong Baptist University, Xianjiang, China; 6 Institute for Research and Continuing Education, Hong Kong Baptist University, Shenzhen, China

**Keywords:** autoimmunity, cytokine, immune regulation, inflammatory disease, signaling

Chronic inflammation, driven by dysregulated cytokine networks, lies at the heart of a vast spectrum of debilitating conditions, from autoimmune disorders like rheumatoid arthritis (RA) and lupus nephritis (LN) to complications like post-surgical fibrosis and recurrent spontaneous abortion (RSA). Despite their prevalence and impact, the intricate molecular choreography governing cytokine signaling in these diseases remains incompletely mapped. This second volume of our Research Topic, *“Unraveling the Molecular Mechanisms of Cytokine Signaling in Regulating Inflammatory Diseases,”* directly confronts this knowledge gap. The compelling research presented herein provides critical mechanistic insights into cytokine pathways, revealing novel regulatory nodes, unexpected cellular dialogues, and promising therapeutic targets, collectively advancing our journey towards precision immunology.

The core mission of this Research Topic – to dissect the molecular mechanisms of cytokine signaling dysregulation and identify pathways for therapeutic intervention – is vividly reflected in the diverse studies published. A central theme is the development of sophisticated strategies to restore immune balance. Qiao et al. exemplify this by demonstrating the remarkable long-term efficacy of combining an αCD4 antibody with a specific retinal antigen (IRBP1-20) in a chronic model of autoimmune uveitis. Their work transcends acute suppression, showing sustained long-term disease control, mediated by induced regulatory T cells (Tregs) and profound downregulation of ocular inflammatory cytokine gene networks. This study underscores the power of targeted immunomodulation to reprogram the local inflammatory microenvironment for durable benefit.

Understanding the specific molecular pathways hijacked in inflammation is paramount for targeted therapy. Wang et al. delve into the complex world of fibrosis, a common and often treatment-refractory outcome of dysregulated healing. They identify Interferon Induced Transmembrane Protein 1 (IFITM1) as a critical regulator of epidural scar hyperplasia post-laminectomy. Their meticulous work unveils a dual mechanism: IFITM1 promotes fibroblast proliferation and activation via the SMAD3 pathway while simultaneously driving adipocyte-to-fibroblast transformation by suppressing the fatty acid synthesis protein CBR4. This intricate crosstalk between IFITM1, SMAD3, and metabolic reprogramming in adipocytes highlights novel targets (IFITM1, CBR4) for preventing pathological fibrosis.

The dialogue between immune cells and tissue-resident cells is another crucial layer of cytokine regulation explored in this Research Topic. Yan et al. shed light on the tragic pathophysiology of RSA by elucidating a devastating positive feedback loop between M1 macrophages (M1-Mφ) and trophoblasts. They identify CXCL9, elevated in RSA decidua, as the key mediator released by M1-Mφ. CXCL9 signals through CXCR3 on trophoblasts, activating JAK/STAT1 and subsequently inducing the transcription factor ZEB1 via IRF1. ZEB1 then drives CCL2 production, recruiting more macrophages and perpetuating inflammation while directly inhibiting trophoblast migration and invasion – essential processes for successful pregnancy. This CXCL9/STAT1/IRF1/ZEB1/CCL2 axis represents a self-amplifying inflammatory circuit and a compelling target for breaking the cycle of RSA.

The integration of metabolism and immune function is increasingly recognized as fundamental. Zhang et al. provide a comprehensive systems-level analysis in RA, integrating transcriptomics and clinical data. They pinpoint dysregulation in amino acid, energy, and TCA cycle pathways as key discriminators of RA. Crucially, they construct a co-expression network linking the TCA cycle to immune infiltration and identify 11 hub genes (including COX7C, validated *in vivo*) that act as molecular bridges between metabolic reprogramming and immune dysregulation. Their LASSO diagnostic model, based on these genes, offers not only diagnostic potential but also a framework for understanding how metabolic shifts fuel synovitis, paving the way for metabolically targeted interventions.

Exploring specific cytokine-mediated regulatory mechanisms, Giraulo et al. investigate the purinergic anti-inflammatory pathway in psoriasis. They demonstrate that the pro-inflammatory cytokine Oncostatin M (OSM), part of the psoriatic “M5 cocktail,” paradoxically upregulates the ecto-enzyme CD73 on keratinocytes via the JAK/MAPK pathway. CD73 generates extracellular adenosine, which then signals primarily through upregulated A2A receptors (A2AR) on both keratinocytes and dermal fibroblasts to suppress IL-8 release. This work reveals a crucial endogenous anti-inflammatory feedback loop (OSM/CD73/Adenosine/A2AR) within the inflamed skin, suggesting that boosting CD73 activity or A2AR signaling could be therapeutic strategies.

Beyond specific diseases, Dong and Yue’s systematic review reframes Obstructive Sleep Apnea (OSA) as a potent driver of systemic immune dysregulation. They synthesize evidence showing how intermittent hypoxia and sleep fragmentation activate master regulators like HIF-1α, NF-κB, and the NLRP3 inflammasome across innate and adaptive immune cells. This chronic immune activation underpins the systemic comorbidities of OSA (cardiovascular, metabolic, cognitive). Their work emphasizes that understanding the immunology of OSA is essential for developing therapies that address its systemic consequences, moving beyond mere airway management.

Finally, robust clinical validation of cytokine pathways is essential. Zhou et al. present a rigorous meta-analysis confirming Interleukin-18 (IL-18), a key pyroptosis-related cytokine, as a significant biomarker in Lupus Nephritis (LN). They demonstrate significantly elevated circulating IL-18 levels in LN patients compared to SLE patients without nephritis and healthy controls, with levels highest in severe Class IV LN. This provides strong clinical evidence for IL-18 signaling dysregulation in LN progression, solidifying its role as a therapeutic target and a biomarker for renal involvement and severity.

Conclusion: Towards Precision Immunomodulation

The collective findings in this volume powerfully illustrate the dynamic complexity and profound clinical relevance of cytokine signaling networks in inflammatory diseases. From unveiling novel axes like CXCL9/STAT1/ZEB1/CCL2 in RSA and OSM/CD73/A2AR in psoriasis, to identifying key regulators like IFITM1 in fibrosis and metabolic hubs like COX7C in RA, these studies provide deep mechanistic insights. They highlight the intricate crosstalk between immune cells, stromal cells, and metabolic pathways, and underscore the importance of biomarkers like IL-18 for patient stratification. Critically, several studies offer tangible therapeutic avenues: inducing antigen-specific tolerance (uveitis), targeting specific signaling nodes (IFITM1, CBR4, CXCL9, STAT1), modulating metabolic pathways, or enhancing endogenous anti-inflammatory mechanisms (CD73/adenosine).

This progress exemplifies the core aim of this Research Topic: moving beyond descriptive phenomenology to a mechanistic understanding that enables targeted intervention. By unraveling the molecular intricacies of cytokine signaling, the research presented here provides the essential foundation for developing the next generation of precise, effective, and durable therapies for patients burdened by chronic inflammatory diseases. The journey towards restoring immune balance continues, guided by these illuminating molecular maps.

